# Identification of the plasma total cfDNA level before and after chemotherapy as an indicator of the neoadjuvant chemotherapy response in locally advanced breast cancer

**DOI:** 10.1002/cam4.2906

**Published:** 2020-02-03

**Authors:** Ge Ma, Jingyi Wang, Huaxing Huang, Xu Han, Jin Xu, Jordee Selvamanee Veeramootoo, Tiansong Xia, Shui Wang

**Affiliations:** ^1^ Department of Breast Surgery The Firsft Affiliated Hospital with Nanjing Medical University Nanjing China; ^2^ Jiangsu Key Lab of Cancer Biomarkers, Prevention and Treatment Jiangsu Collaborative Innovation Center for Cancer Personalized Medicine School of Public Health Nanjing Medical University Nanjing China; ^3^ The First Clinical Medical College Nanjing Medical University Nanjing China; ^4^ Department of Breast and Thyroid Surgery Nanjing First Hospital Nanjing Medical University Nanjing China

**Keywords:** apoptosis, biomarkers, breast cancer, neoadjuvant chemotherapy

## Abstract

This study aimed to retrospectively evaluate the circulating free DNA (cfDNA) level in patients with locally advanced breast cancer (LABC) having different neoadjuvant chemotherapy (NCT) responses and to investigate whether dynamic changes in cfDNA level could predict the effectiveness of NCT in patients with LABC. Data on 61 patients with LABC were included. NCT responses were evaluated using the response evaluation criteria. Blood samples were collected for cfDNA detection before treatment and after the first and eighth courses of chemotherapy. The Alu 111‐bp and 260‐bp fragment levels were evaluated by polymerase chain reaction, and the predictive value of the cfDNA level in the NCT response was determined. In vitro, the MCF‐7 and MCF‐7/ADR cell lines were applied to simulate the phenomenon of drug resistance and explain the underlying mechanism. The Alu 111‐bp level increased after the first NCT course (*P* = .014) and then remained high after NCT in the high‐R group (*P* = .047), but it remained steady in the low‐R group during NCT. A similar tendency in the Alu 260‐bp level was revealed in different groups. The ∆∆Ct value of Alu 260‐bp had good diagnostic efficiency in assessing predictive ability. The area under the curve for the ∆∆Ct1 and ∆∆Ct2 of Alu 260‐bp was 0.697 and 0.647, respectively. The cfDNA level was closely related to epirubicin‐induced apoptosis and changes in the Ki‐67 index in vitro. The elevation of cfDNA after one chemotherapy cycle was mediated by the apoptosis of tumor cells and related to the improved chemotherapy response.

## Graphical abstract

The cfDNA level was closely related to epirubicin‐induced apoptosis and changes in the Ki‐67 index. The elevation of cfDNA after one chemotherapy cycle was mediated by the apoptosis of tumor cells and related to the improved chemotherapy response.

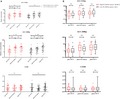

## 1 INTRODUCTION

Breast cancer is the most common malignant disease in women.^1^ Neoadjuvant chemotherapy (NCT) plays an important role in locally advanced breast cancer (LABC). However, the response of patients to chemotherapy largely varies. NCT increases the chances of surgery. At the same time, chemotherapy resistance may affect the survival outcomes by delaying the treatment time. Hence, simple and efficient predictors of the NCT response need to be urgently explored.

The traditional method with human plasma was found to have low diagnostic efficiency. Consequently, liquid biopsy based on the analysis of tumor components in bodily fluids received increasing attention. Tumor components include circulating tumor cells and circulating tumor nucleic acid substances.^2^

Circulating free DNA (cfDNA) is detected in not only patients with cancer but also in healthy individuals.^3^ Most of the studies focused on the diagnostic value of cfDNA in early‐stage neoplastic diseases. The plasma levels of cfDNA were significantly higher in patients with breast cancer than in patients with benign tumors.^4^, ^5^, ^6^ The analysis of cfDNA could be used as a surrogate for tissue biopsies. However, a few studies tried to explore the correlations between cfDNA and treatment response. Individual studies with a small sample size provided inconsistent information on the predictive value of cfDNA in patients undergoing NCT.^7^, ^8^ The conclusions could be skewed by the low homogeneity of participants and small sample size.

In this study, patients with a similar tumor stage were recruited and underwent the same NCT based on anthracycline and taxane drugs. At the same time, the study design of self‐observation before and after the therapy decreased the effect of influencing factors. The findings of this study might offer some important insights into the cfDNA from a new perspective and explore the future prospects for liquid biopsy in NCT.

## 2 METHODS

### 2.1 Patients and sample collection

Between 2016 and 2018, 61 patients diagnosed with LABC were enrolled at the First Affiliated Hospital of Nanjing Medical University. All patients were confirmed to have breast cancer by core biopsy. All patients were staged in LABC. The uniform NCT regimen was EC ×4 to T ×4 [epirubicin (EPI) 90 mg/m^2^, intravenous (iv), d1; cyclophosphamide 600 mg/m^2^, iv, d1; 4 cycles with each cycle of 21 days; and then docetaxel 80 mg/m^2^, iv, d1; 4 cycles with each cycle of 21 days]. Peripheral blood samples were collected before chemotherapy and after the first and eighth courses of chemotherapy. The NCT response classification referred to the Miller‐Payne system after the surgery. The patients with Miller‐Payne 1‐2 grades were classified as the low‐response (low‐R) group, and patients with 3‐5 grades were considered as the high‐response (high‐R) group. The Ki‐67 index values from the postoperative and preoperative biopsy pathology reports were compared and used to evaluate the NCT response. Compared with the basal value of the Ki‐67 index of 66.67% prior to NCT, a higher Ki‐67 index after NCT was considered as low‐R (Ki‐67 mode) and a lower Ki‐67 index was considered as high‐R (Ki‐67 mode).

### 2.2 Plasma DNA isolation

Blood samples were collected for cfDNA detection before the treatment (at the time of biopsy) and after the first and eighth courses of chemotherapy. cfDNA was extracted from 200 µL of plasma using a QIAamp DNA Blood Mini Kit according to the kit protocol, and the final eluate was collected and stored at –20°C. The samples from different groups were always extracted together to avoid batch effects.

### 2.3 cfDNA level and CFDI

The Alu repetitive element was used to estimate the cfDNA level. For this element, 111‐bp and 260‐bp fragments were measured in triplicate using absolute quantitative analysis. The genomic DNA isolated from the SUM‐1315 cells in the culture was used as a reference to determine the relative DNA integrity in plasma DNA. The Ct value of the 111‐bp fragment for a sample was subtracted from that for the SUM‐1315 control to obtain a ΔCt value for the fragment. Similarly, the Ct value of the 260‐bp fragment for the sample was subtracted from that for the SUM‐1315 control to obtain a ΔCt value for the fragment. The following equation was used to calculate CFDI: CFDI = (ΔCt value for 260‐bp fragment –ΔCt value for 111‐bp fragment) × LN 2.^9^

### 2.4 Quantitative reverse‐transcription polymerase chain reaction

cfDNA levels were derived by analyzing the Alu repetitive element. The Alu 111‐bp polymerase chain reaction (PCR) fragment was amplified using the forward primer 5′‐CTGGCCAACATGGTGAAAC‐3′ and the reverse primer 5′‐AGCGATTCTCCTGCCTCAG‐3′; the Alu 260‐bp fragment was amplified using the forward primer 5′‐ACGCCTGTAATCCCAGCA‐3′ and the reverse primer 5′‐CGGAGTCTCGCTCTGTCG‐3′.

The reaction mixture for the qPCR comprised the following: 0.2 µL of 10 µmol/L of each PCR primer, 10 µL of 2× SYBR Green, 1 µL of DNA template, and 8.6 µL of distilled water. The PCR conditions were 10 minutes at 95°C and 40 cycles of denaturation at 95°C for 15 seconds, annealing at 60°C for 60 seconds, and extension at 72°C for 15 seconds using the Roche LightCycle 480 system (Roche Applied Sciences).

### 2.5 Cell lines and culture

The human breast cancer cell line MCF‐7 and MCF‐7/ADR were purchased from ATCC (VA, USA) and cultured in Dulbecco's modified Eagle's medium supplemented with 10% fetal bovine serum, 1% penicillin, and 1% streptomycin. All cells were incubated at 37°C in a humidified chamber supplemented with 5% CO_2_. In vitro*,* the breast cancer cell microenvironment was simulated with EPI. The MCF‐7 and MCF‐7/ADR were exposed to EPI with a gradient concentration of 1‐32 μg/mL for 48 hours. cfDNA was extracted from 200 µL of supernatant.

### 2.6 Flow cytometry measurement of apoptosis and Ki‐67 index

Flow cytometry (FC) was used for analyzing cell apoptosis and quantifying the expression of Ki‐67.

Cell apoptosis was analyzed by applying a cellular staining protocol with an APC Annexin V/Dead Cell Apoptosis Kit containing APC Annexin V and SYTOX Green (Invitrogen, Catalog no. V35113). Ki‐67 was quantified using a classic cellular fixation and permeabilization protocol with 70% ethanol and chilling to –20°C. A mouse IgG anti‐Ki‐67, conjugated with Brilliant Violet 421 dye, was applied for FC analysis. The MCF‐7 or MCF‐7/ADR cell line were treated with an EPI gradient concentration of 0‐32 μg/mL culture medium for 48 hours. The cells were resuspended in the binding buffer at a concentration of 1 × 10^6^ cells/mL. The samples were vortexed and incubated in the dark at room temperature for 30 minutes. After incubation, the cells were analyzed by FC.

### 2.7 Statistical analysis

The levels of all measured markers (both Alus, DNA integrity) were analyzed by repetitive‐measurement deviation analysis. The multiple comparison analysis corrected by the Tukey's test was used to analyze the differences within the groups.

For the ∆Ct of Alu111 and Alu260, the receiver operating characteristic (ROC) curves were generated at three time points. The ∆∆Ct, defined as the difference between the two measurements, was also analyzed using the ROC curve. All statistical analyses were performed using SPSS version 21.0 (SPSS, IBM), and a *P* value <.05 was considered statistically significant.

## 3 RESULTS

### 3.1 Analysis of cfDNA in patients classified by different characteristics

The clinicopathological features of patients and their association with the cfDNA level are shown in Table 1. In groups with different clinical parameters, significant differences in the levels of Alu111 and Alu260 were found at different time points. However, no significant differences in the cfDNA level was observed between the groups with different clinical features.

**Table 1:** Expression levels of the Alu 111‐bp and Alu 260‐bp fragments in patients with different clinical characteristics (presented as mean ± SD; ΔCt)

Factors	111			260			111		260	
	Pre‐NCT	Post‐first NCT	Post‐NCT	Pre‐NCT	Post‐first NCT	Post‐NCT	*P* value^1^	*P* value^2^	*P* value^1^	*P* value^2^
Total										
Age (y)							<.0001	.6394	<.0001	.6577
<50	7.773 ± 1.655	6.178 ± 1.889	6.603 ± 1.676	9.207 ± 1.394	7.583 ± 1.510	7.595 ± 1.681				
≥50	7.865 ± 1.601	6.984 ± 2.082	6.193 ± 1.651	9.031 ± 1.608	8.407 ± 1.755	7.329 ± 1.708				
HER‐2 status							<.0001	.3984	<.0001	.3063
Negative	7.849 ± 1.693	6.765 ± 2.096	6.538 ± 1.652	9.124 ± 1.557	8.195 ± 1.835	7.606 ± 1.840				
Positive	7.780 ± 1.494	6.382 ± 1.910	6.085 ± 1.674	9.082 ± 1.450	7.771 ± 1.392	7.165 ± 1.371				
Molecular subtype							.0002	.8669	.0005	.1482
Hormone + HER‐2–/+	7.859 ± 1.549	6.566 ± 1.953	6.344 ± 1.610	9.051 ± 1.630	7.850 ± 1.604	7.298 ± 1.648				
TNBC	7.533 ± 1.971	6.959 ± 2.401	6.845 ± 1.600	9.487 ± 1.130	8.781 ± 2.067	8.092 ± 1.815				
Hormone‐HER‐2+	8.100 ± 1.573	6.462 ± 2.114	5.737 ± 2.171	8.839 ± 1.195	8.099 ± 1.420	7.360 ± 1.762				
Lymph node							<.0001	.4929	<.0001	.3538
≤2	7.483 ± 1.929	6.724 ± 2.229	6.235 ± 1.758	9.178 ± 1.583	8.290 ± 1.838	7.560 ± 1.701				
>2	8.114 ± 1.242	6.545 ± 1.862	6.493 ± 1.591	9.051 ± 1.462	7.832 ± 1.549	7.351 ± 1.696				

Note

*P* value^1^: time.

*P* value^2^: different clinical characteristics.

### 3.2 cfDNA levels in patients with different chemotherapy effect

A total of 61 patients were divided into groups according to the chemotherapy response in two different evaluation systems.

#### 3.2.1 Patients grouped using the Miller‐Payne system

**Figure 1:**
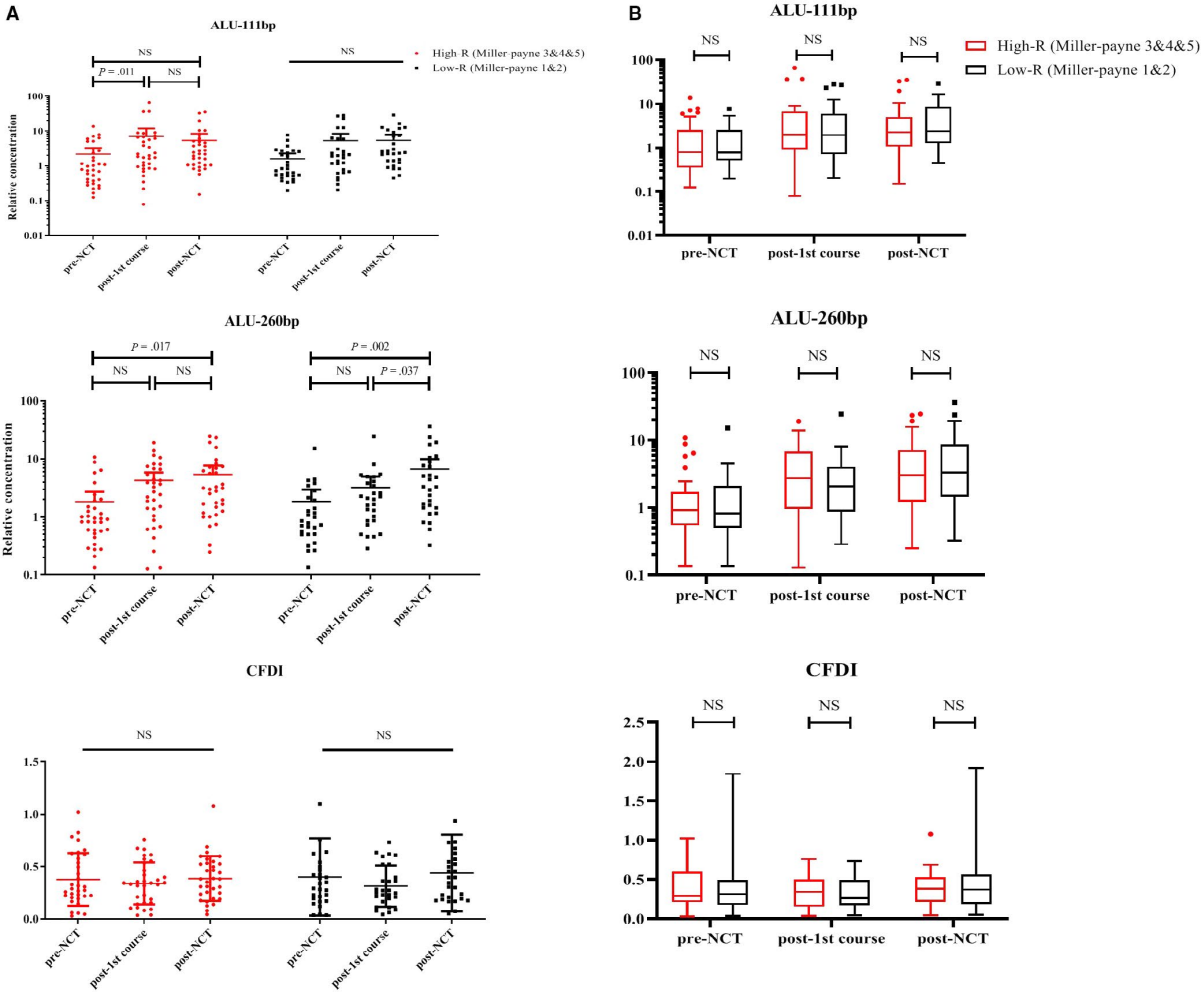
Correlation between cfDNA levels and patients with different Miller‐Payne grades. A, The Alu 111‐bp fragment levels increased after the first NCT in patients with Miller‐Payne 3‐5 grades. The Alu 111‐bp fragment levels remained stable during NCT in the low‐R group compared with the high‐R group. The Alu 260‐bp fragment levels increased after the NCT in both groups. CFDI remained at a constant level. B, No significant difference in the levels of both Alus and CFDI was found between different groups during the whole NCT

According to the Miller‐Payne system, 28/61 patients achieved <30% loss of tumor cells (6/61 grade 1 and 22/61 grade 2), and the other 33 patients were grouped in grade 3‐5 (23, 3, and 7 for grades 3, 4, and 5, respectively). The NCT response according to the Miller‐Payne system had no correlation with age, HER‐2 status, molecular subtype, or lymph node metastasis in this study (Table 2). During NCT, a significant increase in the Miller‐Payne 3‐5 grades was observed after the first NCT course compared with the Alu111 level before NCT (*P* = .011). No significant difference in Miller‐Payne 1‐2 grades was found after the first NCT course. The Alu 260‐bp fragment levels increased after the eighth course in both the groups. CFDI remained steady during the whole course of NCT (Figure 1).

**Table 2:** Relationship between patients with different responses and their clinical characteristics (according to the Miller‐Payne system)

Factors	Total (No.)	High‐R (No.)	Low‐R (No.)	*P* value
Total	61	33	28	
Age (y)				.406
<50		13	14	
≥50		20	14	
HER‐2 status				.262
Negative		19	20	
Positive		14	8	
Molecular subtype				.642
Hormone + HER‐2–/+		22	22	
TNBC		7	4	
Hormone‐HER‐2+		4	2	
Lymph node				.939
≤2		15	13	
>2		18	15	

#### 3.2.2 Patients grouped by changes in the Ki‐67 index

**Figure 2:**
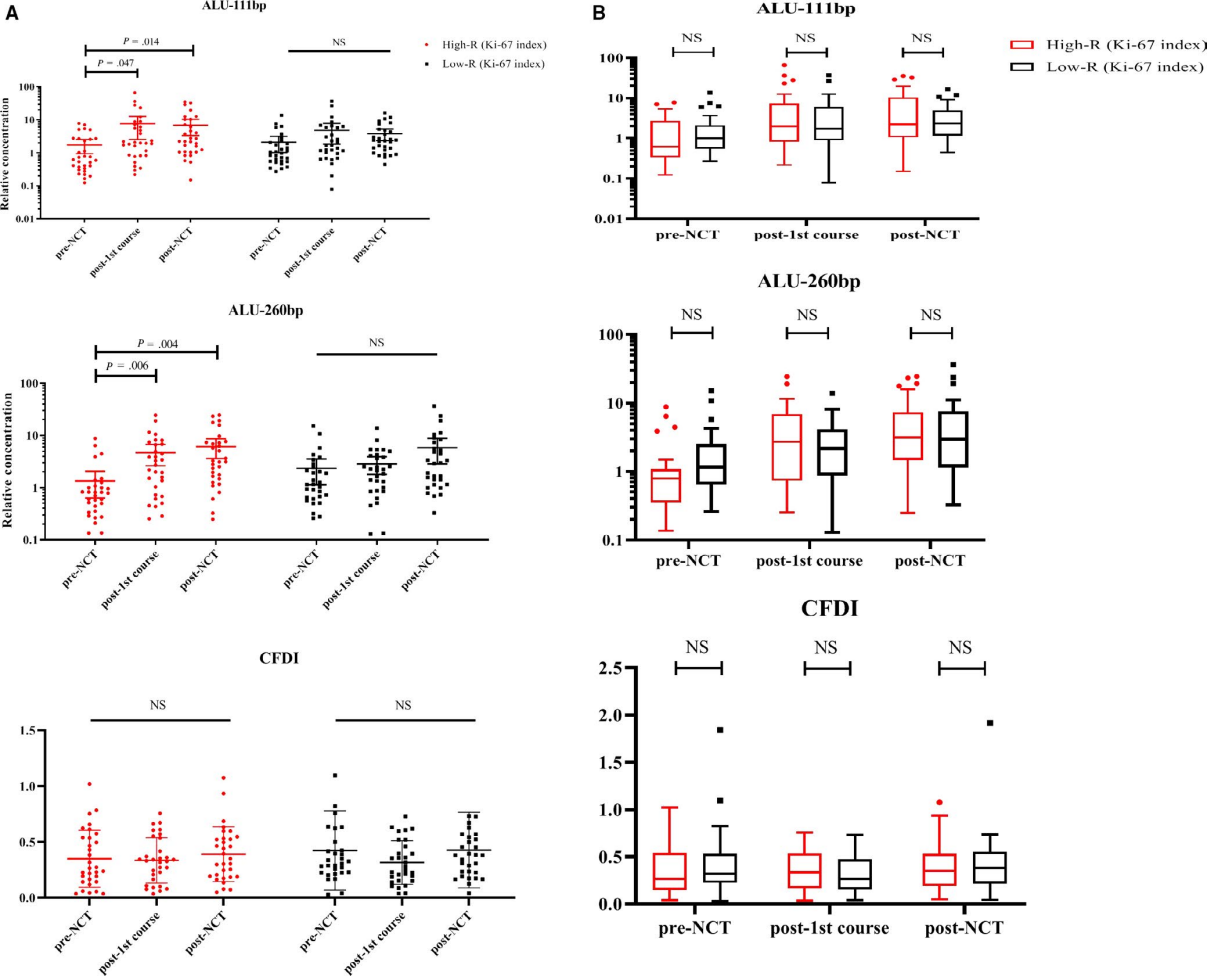
Correlation between cfDNA levels and patients with different changes in the Ki‐67 index. A, The levels of both Alus increased after the first and the eighth NCT in the high‐R group. The cfDNA level remained stable during NCT in the low‐R group. No significant difference was observed during NCT in any of the groups. B, No significant difference in both Alus and CFDI was found between different NCT response groups during NCT

Another grouping method was according to the change in the tumor Ki‐67 index before and after NCT. Furthermore, 30/61 patients (49.18%) had an up to 33.33% decline in the Ki‐67 index (low‐R group), and 31/61 (50.82%) achieved more than 33.33% decrease compared with the biopsy sample after the surgery (high‐R group). Under this evaluation system, there was also no significant relation between the NCT response and the clinicopathological factors of age, HER‐2 status, molecular subtype, or lymph node metastasis (Table 3). But Alu111 increased after the first NCT cycle (*P* = .014) and then remained at a high level after NCT in the high‐R group (*P* = .047). However, Alu111 remained steady in the low‐R group during NCT. A similar tendency of changes in the Alu260 levels was observed in different response groups. No significant differences of CFDI was found in three tests (Figure 2).

**Table 3:** Relationship between patients with different responses and their clinical characteristics (according to the change in the Ki‐67 index before and after NCT)

Factors	Total (No.)	High‐R (No.)	Low‐R (No.)	*P* value
Total	61	31	30	
Age (y)				.240
<50	27	16	11	
≥50	34	15	19	
HER‐2 status				.332
Negative	39	18	21	
Positive	22	13	9	
Molecular subtype				.558
Hormone + HER‐2–/+	44	24	20	
TNBC	11	4	7	
Hormone‐HER‐2+	6	3	3	
Lymph node				.154
≤2	28	17	11	
>2	33	14	19	

### 3.3 Predictive value of the cfDNA level in the NCT response

The three times ∆Ct values of Alu111 and Alu260 were analyzed using ROC curves. The diagnostic efficiency of cfDNA was evaluated using ROC curves in two different evaluation systems. When the Ki‐67 index was taken as the standard, the cfDNA level demonstrated greater predictive power in NCT.

**Figure 3:**
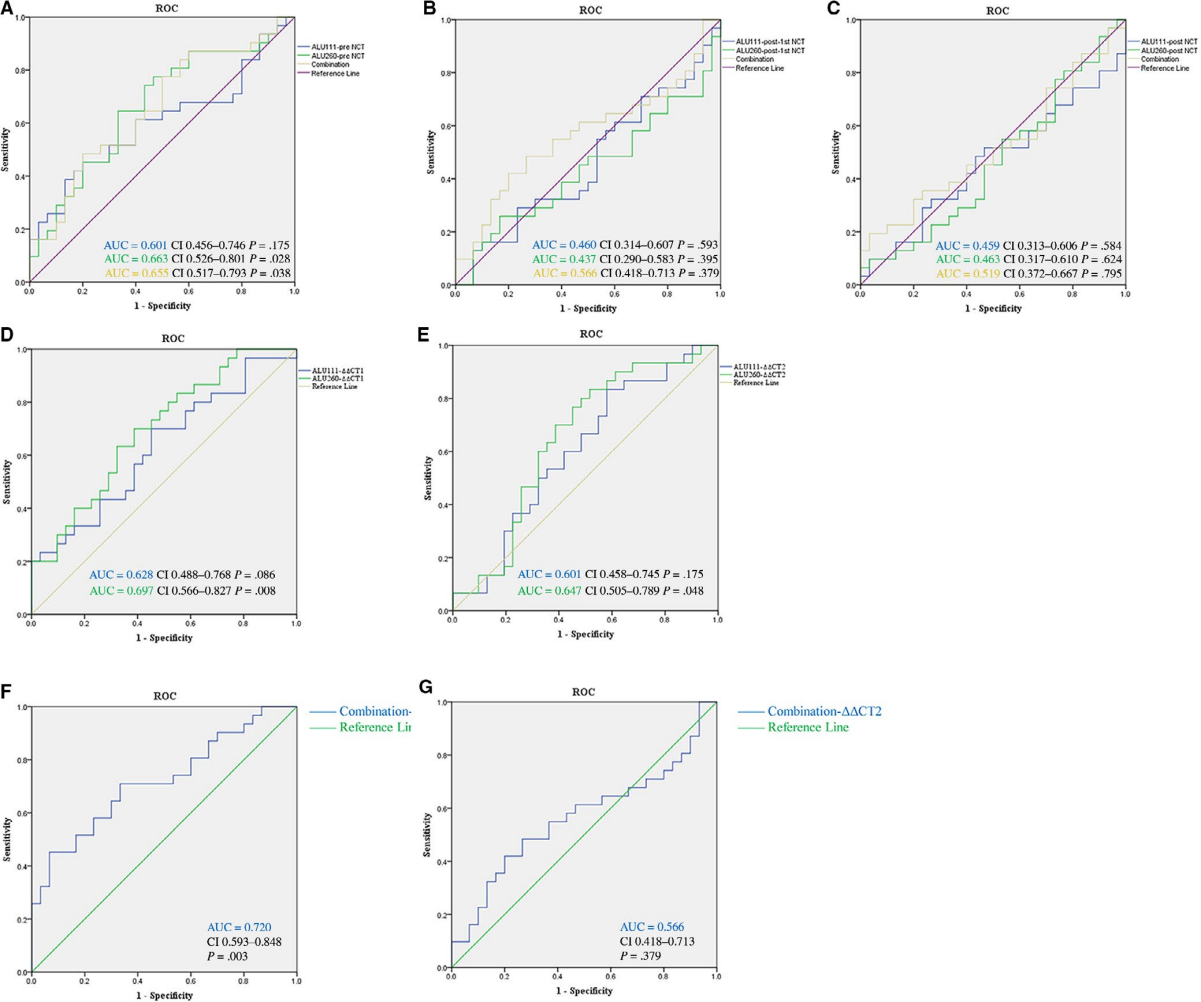
Receiver operating characteristic (ROC) curves for cfDNA for discriminating the low‐response group from the high‐response group. AUC, Area under the curve. A, Relative levels of both Alus and a combined result before NCT. B, Relative levels of both Alus and a combined result after the first NCT. C, Relative levels of both Alus and a combined result after NCT. D, The ∆∆Ct1 (the difference between the ∆Ct at the first two points) of both Alus. E, The ∆∆Ct2 (the difference between the ∆Ct at the beginning and the end time points). F, Combined results of the ∆∆Ct1. G, Combined results of the ∆∆Ct2

Figure 3 shows the ROC curves of the cfDNA levels for predicting the NCT response. The area under the curve (AUC) for the Alu111 and Alu260 levels before NCT was 0.601 [95% confidence interval (CI) 0.456‐0.746) and 0.663 (95% CI 0.526‐0.801), respectively. However, the efficacy of the cfDNA levels tested at the second and third time points was considerably less.

∆∆Ct1 was defined as the difference between the ∆Ct at the first two points, and ∆∆Ct2 means the difference between the ∆Ct of the beginning and the end time points. The ∆∆CT value of Alu260 was found to have good diagnostic efficiency in the present study. The AUC for the ∆∆Ct1 and ∆∆Ct2 of Alu260 was 0.697 (95% CI 0.566‐0.827, *P* = .008) and 0.647 (95% CI 0.505‐0.789, *P* = .048), respectively.

The ∆Ct values of Alu111 and Alu260 were used to construct three panels. The equations for the three time points were as follows:

$$\begin{array}{cc} \text{Logit}\left(P1\right) & =-0.89\times \text{Alu}111\left(\text{pre}-\text{NCT}\right)\\ & \;+0.468\times \text{Alu}260\left(\text{pre}-\text{NCT}\right)-3.542 \end{array}$$

$$\begin{array}{cc} \text{Logit}\left(P2\right) & =0.058\times \text{Alu}111\left(\text{post}-\text{first}\;\text{NCT}\right)\\ & \;-0.214\times \text{Alu}260\left(\text{post}-1\text{st}\;\text{NCT}\right)+1.366 \end{array}$$

$$\begin{array}{cc} \text{Logit}\left(P3\right) & =-0.169\times \text{Alu}111\left(\text{post}-\text{NCT}\right)\\ & \;+0.079\times \text{Alu}260\left(\text{post}-\text{NCT}\right)-0.527 \end{array}$$

The panels were also composed using the ∆∆Ct1 or ∆∆Ct2 values of Alu111 and Alu260. The equation for ∆∆Ct1 was as follows: Logit(∆P1) = 0.341 × Alu111 (∆∆Ct1) – 0.741 × Alu260 (∆∆Ct1) – 0.345; and the one for ∆∆Ct2 was as follows: Logit(∆P2) = – 0.27 × Alu111 (∆∆Ct2) – 0.218 × Alu260 (∆∆Ct2) – 0.292.

All these panels were used to predict the probability of NCT responses by the logistic regression model. It showed the highest diagnostic efficiency in ∆*P*1. The AUC was 0.720 (CI 0.593‐0.848, *P* = .003).

### 3.4 EPI‐induced Cell apoptosis led to an increase in the cfDNA levels

**Figure 4:**
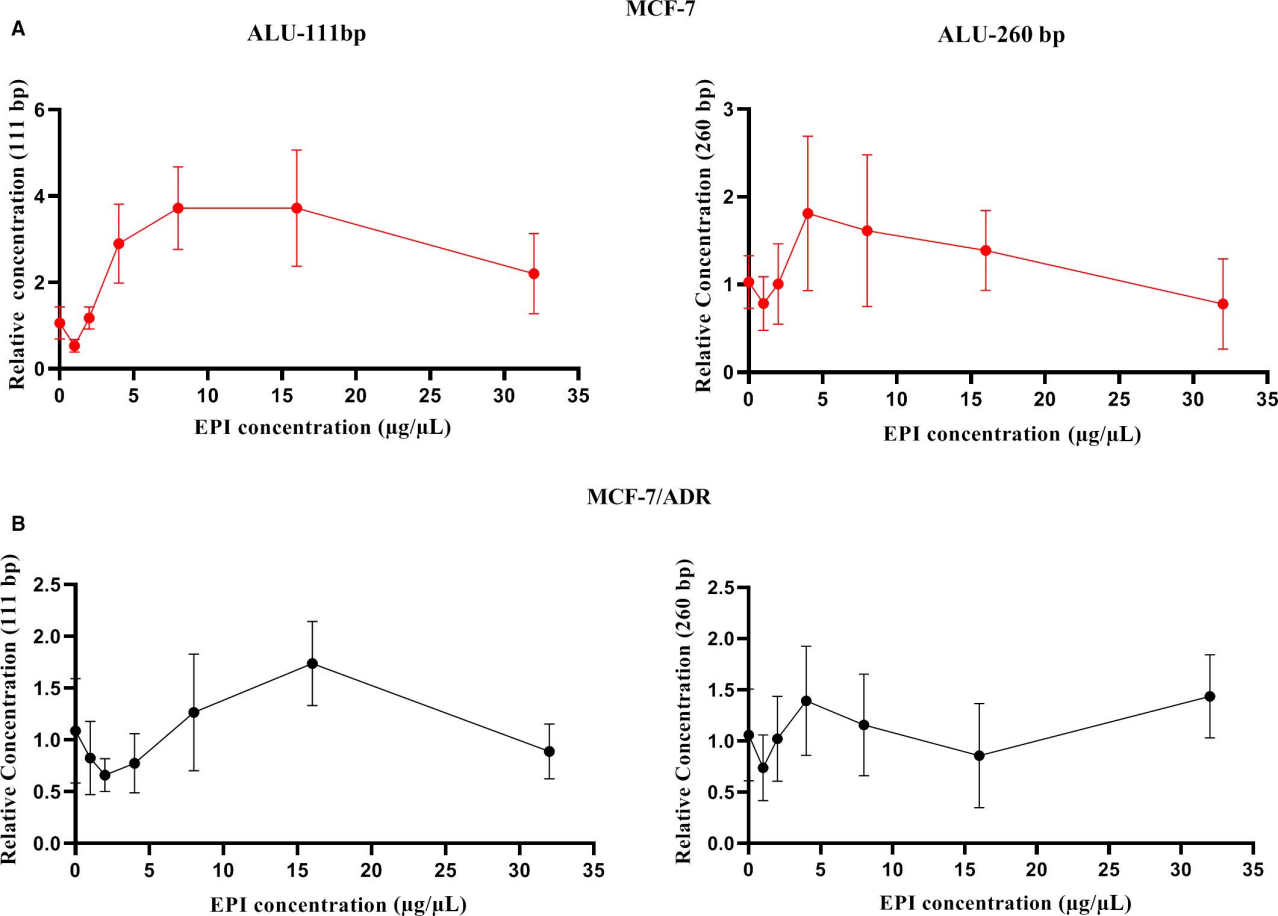
The cfDNA levels in the supernatant after the treatment of epirubicin. A, A decrease and then increase in the Alu 111‐bp fragment levels in the supernatant was observed in both the MCF‐7 and MCF‐7/ADR cell lines. The Alu 111‐bp fragment levels decreased at the EPI concentration less than 1 μg/mL in the MCF‐7 cell line. The critical value was 4 μg/mL in the MCF‐7/ADR cell line. B, The Alu 260‐bp fragment levels decreased at the EPI concentration less than 2 μg/mL and then increased in the MCF‐7 cell line. No obvious change in the Alu 260‐bp fragment levels at different EPI concentrations was observed in the MCF‐7/ADR cell line

The MCF‐7 and MCF‐7/ADR cell lines were treated with the EPI gradient concentration in the culture medium. In MCF‐7, the Alu111‐bp fragment was inhibited at a low concentration (<1 μg/mL) and promoted at a higher concentration (1‐16 μg/mL) of EPI. With the increase in the EPI concentration, the Alu 111‐bp fragment remained steady or declined slightly. The MCF‐7/ADR cell line was tested in vitro using simulated tumors with chemo‐resistance. Figure 4 presents a slightly declining trend of the Alu111‐bp fragment in the supernatant until the EPI concentration was 4 µg/mL; at a higher concentration of EPI, the Alu 111‐bp fragment also had a significant elevation (8‐16 μg/mL). The change in the Alu 260‐bp fragment was almost the same in the MCF‐7 and MCF‐7/ADR cell lines with the increase in the EPI concentration.

**Figure 5:**
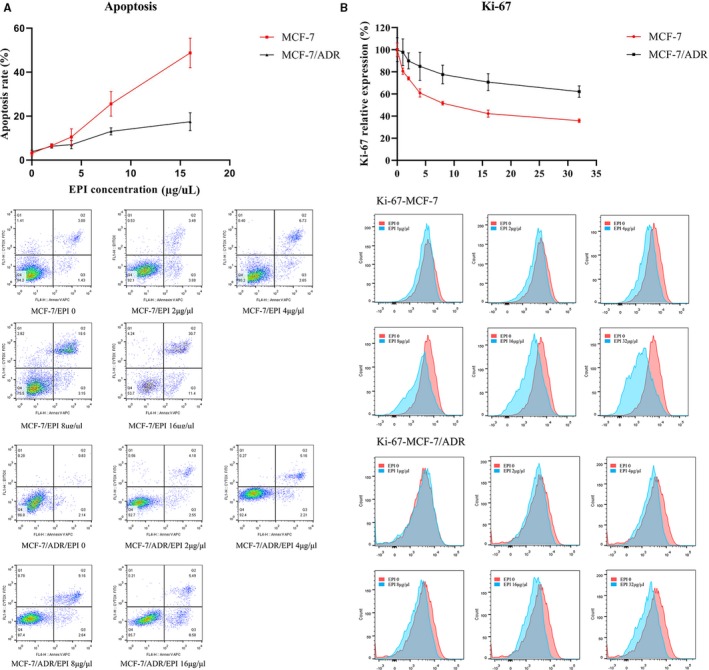
A, The cell apoptosis rate increased with the increasing concentration of EPI in the supernatant in the MCF‐7 cell line. The apoptosis rate was steady at the low concentration gradient of EPI and increased obviously until the concentration was 8 μg/mL in the MCF‐7/ADR cell line. B, The Ki‐67 index decreased with the increase in the EPI concentration in the MCF‐7 cell line. The Ki‐67 index showed minimal change in the MCF‐7/ADR cell line

Figure 5A shows the apoptosis rate of cell lines with different EPI sensitivity. FC showed that the cell apoptosis rate increased with increasing concentrations of EPI in the supernatant in MCF‐7 cells. At the same time, the Alu111 level increased gradually. In the MCF‐7/ADR cell line, the apoptosis rate was steady at the low concentration gradient of EPI and increased obviously until the concentration was 8 μg/mL. The Ki‐67 index of the MCF‐7 and MCF‐7/ADR cell lines was tested using FC and EPI gradient concentration (Figure 5B). The Ki‐67 index decreased with the increase in the EPI concentration in MCF‐7 cells. Similar changing trends were not evident in the MCF‐7/ADR cell line.

## 4 DISCUSSION

cfDNA was released by both active and passive approaches. All living cells may actively release DNA fragments.^10^, ^11^ The passive release may be attributed to apoptosis and necrosis.^12^, ^13^ cfDNA may be released from primary tumor cells, CTCs, latent micro‐metastatic foci, and normal cells. The cfDNA in healthy controls primarily derive from the apoptosis of lymphocytes and other nucleated cells in the peripheral blood. In the patients with neoplastic diseases, the hypoxic‐ischemic necrosis in the center of the tumor may significantly enhance the release of long DNA fraction, besides the release of DNA fraction from normal and diseased cells.^13^, ^14^ Previous studies showed that different fragments were used to differentiate between apoptotic and necrotic cell death.^7^, ^15^, ^16^ Apoptotic cells often release short DNA fragments (Alu 111‐bp fragment) while the longer Alu 260‐bp fragments are considered the products of necrosis.^16^

In most clinical studies, cfDNA was suggested to be a promising diagnostic indicator.^6^, ^17^, ^18^ In addition, several reports on different cancers ascribed a prognostic value of cfDNA measurements to disease‐free or overall survival.^19^, ^20^ However, data on the prediction and monitoring of therapy response in cancer patients undergoing NCT are limited.^21^ In the present study, 2 Alu fragments, 111 bp and 260 bp, were tested in patients with LABC undergoing NCT for their relevance in the pretherapeutic prediction and the intratherapeutic monitoring of the tumor response to treatment.

Neoadjuvant chemotherapy, which is the standard of care for patients with locally advanced and inflammatory breast cancer, has become increasingly popular. Despite a certain lag, NCT is still an effective treatment modality to identify drug sensitivity or biomarker validity. The patients enrolled in the present analysis were in the similar TNM stage. In addition, all patients received the same chemotherapy regimen, and the blood sample collection was at the same points. Based on these homogeneous samples, the difference between patients with low and high responses was more persuasive. However, all patients receiving NCT displayed a continuous pattern, from low response to high response, just like a continuous spectrum of the sunlight. The group standard of treatment response is always the key point. It can classify methods for estimating chemotherapy responses after NCT. Even the accepted standard, a complete pathological response, has some disputes in its definition.

The Miller‐Payne system was developed in 2003 and generally accepted by oncologists.^22^ In this study, 37.7% of all patients were in the Miller‐Payne 3 grade, which was defined as “between an estimated 30% and 90% reduction in tumor cells.” To ensure the specificity of chemotherapy‐resistant cases, the grade 3 cases were classified into the high‐R group. Ki‐67 is a nuclear protein indicating the proliferative activity of tumor cells. The changes in proliferation are a prerequisite for the changes in tumor growth rate and a fall in the Ki‐67 proliferation index during NCT, predicting long‐term benefits. In this study, a comparison of the Ki‐67 index before and after NCT was used as another standard to distinguish the high and low responses. This standard reflected the drug susceptibility from a new perspective. The chemotherapy response prediction was the major objective in the present study. The change in the Ki‐67 index is an effective indicator to distinguish different tumor cell sensitivities to chemotherapy.

In the model of chemotherapy response prediction, the cfDNA level had a similar varying tendency under different grouping schemes. The measured results of PCR revealed no difference in the cfDNA level between high‐R and low‐R groups at all three time points. The self‐matching test showed that the changing trends for different response groups were opposite. The Alu 111‐bp fragment levels, which is an indicator of apoptosis, remained relatively stable during NCT in the low‐R group. However, the Alu 111‐bp fragment levels increased significantly to a variable extent in different periods in the high‐R group. The self‐controlled comparison before and after treatment could eliminate the effect of individual differences at the baseline level and highlight the different impacts of chemotherapy on different patients. Based on the results of the present study, it was hypothesized that the elevated cfDNA levels after one course of chemotherapy were related to the improved response. Apoptosis‐induced passive secretion may be the source of cfDNA. CFDI was also evaluated in the study. No significant difference in the cfDNA level was observed between the high‐R and low‐R groups. In the present study, the apoptosis of the hematopoietic cells induced by chemotherapy was an inevitable question. The concentration of DNA fragments (ALUs) in plasma was tested in our study. In those patients who respond to chemotherapy, the concentration of ALU111‐bp elevated dramatically. But in patients with chemoresistance, the ALU111‐bp level kept stable, which suggested the contribution of the apoptosis of hematopoietic cells in plasma cfDNA was transient and limited.

A few recent studies focused on cfDNA level in patients with LABC undergoing NCT. A study performed by Lehner et al suggested that patients with a better response had a significant decrease in the cfDNA level before the sixth cycle of NCT.^7^ Compared with the aforementioned two similar studies, the present study reached an entirely different conclusion. The differences might be related to different blood collection points and different chemotherapy options. Another study on patients with locally advanced rectal cancer reported a correlation between a high baseline plasma level of cfDNA and an increased risk of recurrence.^8^ It was believed that the high cfDNA level at the baseline was often accompanied by later tumor staging or a greater tumor load.

The priority of the present study was the similar TNM stage and the same regimen of chemotherapy. Furthermore, two different grouping modes ensured the reliability of the conclusions. The increase in the cfDNA level was attributed to the DNA peak after treatment, which was proved by previous studies.^23^, ^24^ In these studies, mouse tumor models were used to evaluate the change in the cfDNA level after treatment, and a spike was observed after chemotherapy. A few previous studies also suggested that an excess of apoptotic cell death, as after chemotherapy, could lead to a saturation of apoptotic cell engulfment and thus increased nucleosome levels in the blood.^25^ The tumor‐free Balb/c mice received chemotherapy was used to evaluate the cfDNA elevation caused by the apoptosis of hematopoietic cells. Our results revealed that the concentration of ALU111‐bp kept steady and slightly elevated until 48 hours after chemotherapy (Data not shown). As a result, the release from the apoptosis tumor cells may explain a larger part of the change of cfDNA.

To confirm this, an in vitro experiment was conducted. In vitro, the breast cancer cell line MCF‐7 and the EPI‐resistant cell line MCF‐7/ADR were used to study the relationship between the cfDNA level and the changes in the biological behavior at different EPI concentrations. As expected, a higher apoptotic rate and a decline in the Ki‐67 index were revealed at high concentrations of EPI in the MCF‐7 cell line. Similar changes in the MCF‐7/ADR cell line were much less significant. The cfDNA level in the supernatant was detected synchronously. The Alu 111‐bp fragment was inhibited at a low drug concentration and released at a high drug concentration. This phenomenon was found in both the MCF‐7 and MCF‐7/ADR cell lines; the only difference between them was the cutoff value of the drug concentration. The critical value of the change was about 1 μg/mL in the MCF‐7 cell line and about 4 μg/mL in the MCF‐7/ADR cell line. It was believed that the relatively low concentration might suppress the active secretion of cfDNA, whereas apoptosis induced by high concentration resulted in the release of cfDNA eventually.

Despite significantly different variations in the cfDNA level before and after NCT between the high‐R and low‐R group, the diagnostic efficacy of cfDNA was generally low. A comprehensive assessment of the changing value of Alu111 and Alu260 between the baseline and post‐first NCT may provide a certain predictive value of efficacy.

In summary, on the prerequisite of the improved homogeneity of participants, the present study compared the different variations in different NCT response groups. The elevation of the cfDNA level after one chemotherapy cycle might be mediated by the apoptosis of tumor cells and related to the improved chemotherapy response.

## CONFLICT OF INTEREST

The authors declare that they have no competing interests.

## AUTHOR CONTRIBUTIONS

Ge Ma contributed to conceptualization, project administration, formal analysis, writing – original draft, and review and editing. Jingyi Wang contributed to project administration and data analysis. Huaxing Huang contributed to data curation and methodology. Xu Han contributed to project administration and data processing. Jin Xu contributed to writing—original draft and review and editing. Jordee Selvamanee Veeramootoo contributed to language editing and data curation. Tiansong Xia contributed to conceptualization and project administration. Shui Wang contributed to conceptualization, supervision, and funding acquisition.

## ETHICS APPROVAL

All procedures were approved by Institutional Review Boards of the First Affiliated Hospital with Nanjing Medical University.

## CONSENT FOR PUBLICATION

All authors have given consent for publication.

## Data Availability

The data that support the findings of this study are available from the corresponding author upon reasonable request.
